# The Effects of Curcumae Longae Radix, Curcuma phaeocaulis Radix and Their Processed Products on Epo/EpoR Pathway and CD62p

**DOI:** 10.3389/fphar.2018.00736

**Published:** 2018-07-09

**Authors:** Zhimin Chen, Wenbing Li, Liang Quan, Haiting Zhou, Yongfeng Zhao, Xi Zhang, Lin Hu, Changjiang Hu

**Affiliations:** ^1^Chengdu University of Traditional Chinese Medicine, Chengdu, China; ^2^Chengdu Institution of Chinese Herbal Medicine, Chengdu, China; ^3^Key Laboratory of Chinese Medicine Formulations Particle Mass and Clinical Evaluation, Chengdu, China; ^4^Neo-Green Pharmaceutical Co., Ltd., Chengdu, China

**Keywords:** Curcumae Radix (Yujin), processing (Paozhi), erythropoietin (Epo), erythropoietin receptor (EpoR), CD62p

## Abstract

**Background:** Curcumae Radix (Yujin) has been widely used to treat Qi stagnation due to Liver depression (LDQS) in traditional Chinese medicine (TCM) for a long time and is good at dispelling melancholy by soothing liver to regulate qi and relieving pain by promoting blood circulation for removing blood stasis. Yujin stir-frying with vinegar can strengthen the effect of dispersing stagnated hepatoqi to stop pain by injecting medicine into the liver and stir-frying with wine can strengthen the effect of promoting blood circulation for removing blood stasis. Because the reason for the enhancement of clinical efficacy by processing is unclear, it is difficult to select and use processed products in the clinic.

**Aim/Hypothesis:** In this study, from the point of view of the platelet function, we start to investigate the mechanism for promoting blood circulation, removing blood stasis, and enhancing clinical efficacy by processing.

**Methods:** This study mainly takes Curcumae Longae Radix (HSYJ) and Curcuma phaeocaulis Radix (LSYJ) as the research subjects. They are genuine drugs in Sichuan Province, China. A high-performance liquid chromatography (HPLC) is used to analyze the main chemical constituents of Yujin and its processed products, and show the difference between the two species, with and without processing. A rat model of blood stasis induced by LDQS is established by giving the tail clamp stimulation, injecting epinephrine hydrochloride subcutaneously, and treating with an effective dose (0.9 g⋅kg^-1^) according to the conversion of human clinical dosage for 2 weeks. After the experiment, the serum levels of erythropoietin are measured by ELISA. Furthermore, RT-PCR and WB are used to detect EpoR mRNA and protein expression in the hepatic tissue. Flow cytometry is used to measure peripheral blood CD62p expression.

**Results:** There is a great difference between the chemical compositions of the two species, the number of chromatographic peaks of Curcumae Longae Radix is more than that of Curcuma phaeocaulis Radix. Curcuminoids is the main component of HSYJ, while curcuminoids is almost free from LSYJ. Curcuminoids is almost insoluble in water. After stir-frying with vinegar or wine, it can increase the dissolution of curcuminoids in water. In rat models, the levels of Epo, EpoR, and CD62p are significantly increased. After intragastric administration of Yujin, indicators show varying degrees of callback. HSYJ is better than LSYJ, and the processed product by stir-frying with wine is better than other processed products.

**Conclusion:** The results show that the mechanism of promoting blood circulation for removing blood stasis in Yujin may be able to inhibit the activation and aggregation of platelets by intervening the Epo/EpoR pathway and regulating CD62p down. Stir-frying with wine can enhance this effect.

## Introduction

Blood stasis is a common clinical syndrome, which can cause coronary heart disease, hypertension, hyperlipidemia, tumor, and many other diseases (Baojun et al., 2013; Guan and He, 2014; He et al., 2016; Zhou et al., 2016). According to TCM, syndrome types of blood stasis include qi deficiency, qi stagnation, blood deficiency, and phlegm obstruction (Liang et al., 2016). Blood stasis induced by LDQS is a common syndrome type, which is caused by unpleasant emotion, or by the liver qi stagnation for a long time caused by the invasion of external evil. It has two pathological states: qi stagnation and blood stasis, which often cause a variety of diseases and harm human health (Liu et al., 2018). In Chinese pharmacopeia, Curcumae Longae Radix (Huangsiyujin, HSYJ) and Curcuma phaeocaulis Radix (Lvsiyujin, LSYJ) in Sichuan can both be used as Yujin, and traditionally the effect of HSYJ is better than LSYJ. Meanwhile, as a common medicine for blood stasis induced by LDQS in TCM clinic, there are a variety of processed products of Yujin, their functions are also dissimilar. Raw Yujin is good at opening despression by soothing liver to regulate qi and relieving pain by promoting blood circulation for removing blood stasis; stir-frying with vinegar can help to introduce medicine into the liver and strengthen analgesic effect by dispersing stagnated hepatoqi; stir-frying with wine can strengthen the effect of promoting blood circulation for removing blood stasis.

Previous studies have shown that Yujin has not only good analgesic effect but also can improve hemorheological state of rats, but its mechanism is not yet clear. Modern studies have shown that Epo is not only associated with blood generation and regulation but also has antidepressant effect (Zhang et al., 2017). Furthermore, Epo-EpoR can also induce activation of platelets and endothelial cells, which contribute to the formation of physiological thrombosis (Heinisch et al., 2012). Platelet activation is closely related to pathology, pathogenesis, diagnosis, and treatment of blood stasis syndrome (Hu et al., 2012).

Therefore, in this study, the effects of HSYJ, LSYJ and their processed products on Epo/EpoR and platelet activation markers CD62p are compared, in order to verify the effect of Yujin and explore the mechanism of processing.

## Materials and Methods

### Chemicals and Reagents

The reference standards of curcumin, demethoxycurcumin, and bisdemethoxycurcumin (purity ≥98%) were purchased from National Institutes for Food and Drug Control (Beijing, China). Furanodienon and furanodiene (purity ≥98%) were purchased from Chengdu Chroma-Biotechnology Co., Ltd. Methanol and acetonitrile (Fisher, United States) were of high-performance liquid chromatography (HPLC) grade. Other reagents were of analytical purity. Water was glass-distilled and filtered through a Milli-Q water purification system (Millipore, Bedford, MA, United States) prior to use.

### Animals

SPF grade Sprague-Dawley rats (200 ± 20 g) were obtained from Chengdu Dashuo Experimental Animal Co., Ltd. (Chengdu, China). Animals were housed in polypropylene cages and were sent to animal room 5 days in advance for acclimatization. (Chengdu University of Traditional Chinese Medicine, Chengdu, China) Besides, they were maintained under controlled conditions (a 12 h light-dark cycle at 22 ± 2°C) on standard pellet diet and water freely. Animal experiments were approved by the Committee of Scientific Research and the Committee of Animal Care of Chengdu University of Traditional Chinese Medicine (Chengdu, China).

### Trail Grouping

Animals were randomly and equally divided into the following nine groups (male and female in half): control group (CG), model group (MG), Curcumae Longae Radix group (SHG), Stir-frying Curcumae Longae Radix with vinegar group (CHG), Stir-frying Curcumae Longae Radix with wine group (JHG), Curcuma phaeocaulis Radix group (SLG), Stir-frying Curcuma phaeocaulis Radix with vinegar group (CLG), Stir-frying Curcuma phaeocaulis Radix with wine group (JLG), and positive group (PoG).

### Medicinal Materials and Reagents

Yujin was purchased from Sichuan Neautus Traditional Chinese Medicine Co., Ltd. and Chengdu Jiankang Pharmaceutical Co., Ltd. (Chengdu, China). The material was authenticated as the dried root of *Curcuma longa* L and *Curcuma phaeocaulis* Val. by Professor Ming Li (Chengdu University of Traditional Chinese Medicine, Chengdu, China). Xuefu Zhuyu Pian (Lot: 170409) was purchased from Weifang Zhongshi Pharmaceutical Co., Ltd. (Shandong, China). Epinephrine Hydrochloride Injection (Lot: 170507) was purchased from Grand Pharma (China) Co., Ltd. (Wuhan, China).

### Preparation of Processed Medicines in Ready-to-Use Forms

Yujin was prepared in ready-to-use forms according to Standard for Processing of Chinese Traditioan Medicine in Sichuan Province (Vision 2015).

#### Stir-Frying Yujin With Vinegar

Took slices Yujin, mixed them with a certain amount of rice vinegar evenly, moistened them thoroughly until the vinegar was absorbed completely, then poured the drugs into a frying container, fried them with mild fire until they became dark yellow, took them out, and laid them to cool. The amount was 100 kg drugs with 15 kg vinegar per.

#### Stir-Frying Yujin With Wine

Took slices Yujin, mixed them with a certain amount of yellow wine evenly, moistened them thoroughly until the wine was absorbed completely, then poured the drugs into a frying container, fried them with mild fire until they became deep yellow, took them out, and laid them to cool. The amount was 100 kg drugs with 10 kg wine.

### Preparation of Yujin Decoction

Decoction was prepared according to Standard for Management of TCM Decocting Room in Medical Institutions and Technical Requirements for Quality Control and Standard Formulation of TCM Granules.

Yujin and its processed products were boiled 30 min after soaking 30 min with nine times water then filtered. The dregs were boiled twice with seven times water and 30 min each time, then filtered and mixed with the previous filtrate. The mixed decoction was concentrated by vacuum concentration (*T* ≤ 50°C). The dosage for rats was 0.9 g⋅kg^-1^ according to the conversion of human clinical dosage.

#### Positive Solution

Xuefu Zhuyu Pian was grinded up to powder and dissolving in water. The dosage for rats was 0.432 g⋅kg^-1^ according to the conversion of human clinical dosage.

### HPLC Analysis

HPLC determinations were performed by an Agilent HPLC 1,200 instrument (Agilent Technologies, Palo Alto, CA, United States), equipped with a diode array detector an autosampler, a column heater, and a Shim-pack GIST C18 (150 mm × 4.6 mm, 5 μm) column, was used. The gradient elution was carried out with acetonitrile as mobile phase A and water as phase B at a flow rate of 1.0 mL/min (0–2 min, 15A%; 2–17 min, 15–40A%; 17–35 min, 40–55A%; 35–55 min, 55–85A%; 55–60 min, 85–95A%; 60–65 min, 95A%). The injection volume was 10 μL and column temperature was set at 30°C. The wavelengths were monitored at 210 and 425 nm. Sample 1 (Powder of Yujin was extracted with 25 mL methanol by ultrasound for 30 min, followed by filtration. For HPLC analysis, the filtrate was filtered through a filter prior to injection) was applied to study the difference of chemical constituents between HSYJ and LSYJ. Sample 2 (Steam the Yujin decoction of 2.6 and dissolve it with methanol, filtered through a filter prior to injection) was applied to study the changes of main chemical constituents before and after processing.

### Animal Model Establishment and Sample Collection

Except the CG, the rest groups replicated blood stasis induced by LDQS model by reference methods (Qin, 2014). Half an hour after giving drugs, animals were given the tail clamp stimulation and maintained 4 min at a time, once every 2 h, four times a day, continuous stimulation for a week. From the second week, the tail stimulation was maintained 5 min each time, stimulated every 1 h, eight times a day, after the fourth tail stimulation 0.6 mg/kg epinephrine hydrochloride (epinephrine hydrochloride and normal saline was 1:3) was injected subcutaneously and re injection after an interval of 4 h, continuous for a week. In CG animals were injected physiological saline and gavaged the same volume of water as the drug groups. When the model was finished, the animals were fasted more than 12 h, but the water was given normally. The samples were taken under anesthesia.

### Determination of Epo in Serum

The rats were anesthetized. Blood was obtained from the abdominal aorta and allowed to clot for 2 h at room temperature, then centrifuged for 10 min at 3,000 rpm. Removed serum and aliquot and store samples at -20°C to avoid repeated freeze-thaw cycles. Epo was detected with Epo-ELISA Kit (Lot: M25016752, Cusabio Biotech Co., Ltd., Wuhan, China).

### Real-Time PCR Assay

The total RNA of samples was extracted by TRIZOL (Lot: 16596-026. United States), and the removal and reverse transcription of genomic DNA were carried out according to PrimeScript RT reagent Kit (Lot: RR047A, Takara Bio, Dalian, China). The details of the reference genes used in the assays were obtained from the NCBI, and Primer Premier was applied to design and screen primers. All the primers were synthesized by Sangon Biotech Co., Ltd. (Shanghai, China) and purified with ULTRAPAGE. The primers were used as follows: β-actin, forward 5′-GAAGATCAAGATCATTGCTCCT-3′ and reverse 5′-TACTCCTGCTTGCTGATCCA-3′; EpoR, forward 5′-TGTTTCTGGGAGGAAGCGGCGAACT-3′ and reverse 5′-ATGGATGATGCGGTGGTAGCGAGGAG-3′. Using Thermo Scientific PikoReal software (Thermo Scientific, United States), CT (Threshold cycle) values of each sample in a PCR process were analyzed. Calculations of the X relative expression level of mRNA were performed using the 2^-ΔΔCT^ method.

### Western Blot Assay

Rat liver tissue samples were thawed in 37°C water and transferred into 2 mL EP tubes. RIPA Lysis Buffer was added in each tube (rat liver tissue: Lysis Buffer = 1:10), then shredded with a small scissors, and cracked on the crushed ice 10 min. Collected lysate and centrifuged for 5 min at 3,000 rpm (*T* = 4°C). The protein concentration of supernatant was determined by BCA Protein Assay Kit (Lot: KGP903, NanJing KeyGen Biotech Co., Ltd, Nanjing, China). The protein lysates were separated by using the 10% SDS-PAGE, electrotransferred onto the polyvinylidene fluoride (PVDF, Hybond Inc, Escondido, CA, United States), and blocked for 2 h. Then, the PVDF membranes were incubated by using the Epo-R antibody (1:1,000, Lot: AF6211, Affinity Biosciences, United States) and anti-beta actin antibody (1:5,000; Lot: ab8226, Abcam), then incubated at 4°C for the night. The membranes were incubated using 1:5,000 Goat Anti-Mouse IgG H&L (HRP) (Lot: ab6721, Abcam Biotech) at room temperature for 2 h. The reactive protein signals were visualized and evaluated by using the enhanced chemiluminescence kit (ECL, Lot: BL520A, Thermo Scientific, United States). Finally, the protein signals were captured and the bands were analyzed by using the ChemiDoc XRS+ System with Image Lab Software (Bio-Rad Laboratories, Hercules, CA, United States).

### Flow Cytometry Assay

Added three times volumes of 1 × RBC Lysis Buffer into fresh anticoagulant blood 300 μL and mixed. The mixed liquid was cracked at room temperature for 2 min before centrifugation for 5 min at 400 g. Removed liquid supernatant and mixed with 5 mL PBS. Removed liquid after centrifugation, mixed with 100 μL PBS, and incubated by using the PE anti-rat CD62p (P-selectin, Lot: 148306, Dakewe Biotech Co., Ltd.) for 30 min at room temperature in dark. Washed it once with PBS, added 500 μL Binging Buffer, and detected by a CytoFLEX FCM analyzed the expression of blood platelet CD62p with CytExpert Software (Beckman Coulter).

### Statistical Analysis

Experimental data were presented as mean ± SD. Statistical analyses were performed with IBM SPSS Statistics 19.0 (IBM, United States) and Graphpad Prism 5 (GraphPad Software, United States). A statistical significance was defined when *P* < 0.05.

## Results

### Results of HPLC Analysis

**FIGURE 1:**
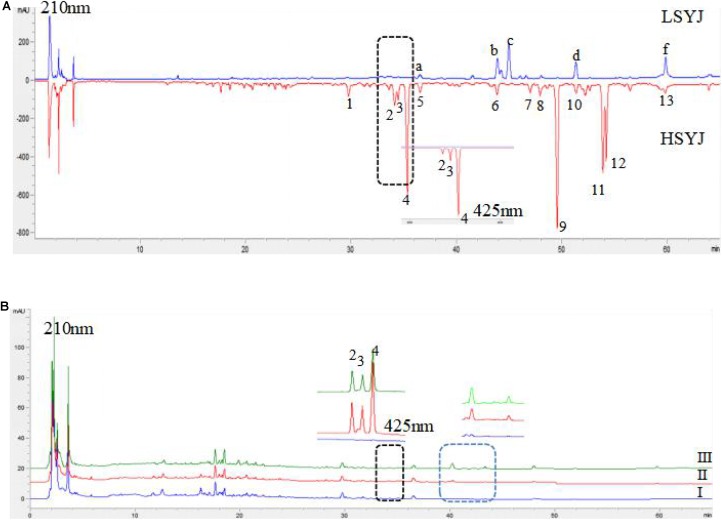
HPLC characteristics spectra of Sample 1 **(A)** and Sample 2 **(B)**. HSYJ, Curcumae Longae Radix; LSYJ, Curcuma phaeocaulis Radix(2) bisdemethoxycurcumin, (3) demethoxycurcumin, (4) curcumin, (c) Furanodienon, (f) Furanodiene, (I) raw, (II) Stir-frying with vinegar, (III) Stir-frying with wine.

There was a great difference in the chemical composition between the two species; the number of chromatographic peaks of HSYJ was more than that of LSYJ. The results are shown in **Figure 1A**. Curcuminoids was the main component of HSYJ, and curcuminoids was almost free from LSYJ. Curcuminoids was almost insoluble in water. After stir-frying with vinegar or wine, it was able to increase the dissolution of curcuminoids in water. The results are shown in **Figure 1B**.

### Epo Level

**FIGURE 2:**
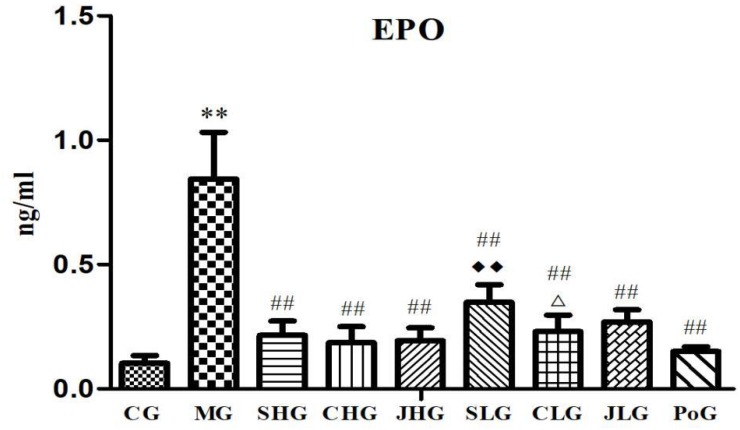
Effect of serum indexes of Epo for various groups of animals. CG, control group, MG, model group, SHG, Curcumae Longae Radix group, CHG, Stir-frying Curcumae Longae Radix with vinegar group, JHG, Stir-frying Curcumae Longae Radix with wine group, SLG, Curcuma phaeocaulis Radix group, CLG, Stir-frying Curcuma phaeocaulis Radix with vinegar group, JLG, Stir-frying Curcuma phaeocaulis Radix with wine group and PoG, positive group. Values were mean ± SD (*n* = 6). ^∗^*p* < 0.05 and ^∗∗^*p* < 0.01 vs. control group; ^#^*p* < 0.05 and ^##^*p* < 0.01 vs. model group; ^Δ^*p* < 0.05 and ^ΔΔ^*P* < 0.01 vs. Curcumae Longae Radix group; ^
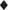
^*P* < 0.05 and ^
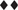
^*P* < 0.01 Curcuma phaeocaulis Radix group versus Curcumae Longae Radix group, Stir-frying Curcuma phaeocaulis Radix with vinegar group versus Stir-frying Curcumae Longae Radix with vinegar group, Stir-frying Curcuma phaeocaulis Radix with wine group versus Stir-frying Curcumae Longae Radix with wine group.

The results of changes of Epo level are shown in **Figure 2**. Compared with the CG, Epo levels in the serum of MG were significantly increased (*p* < 0.01). After the Yujin treatment, the Epo levels of MG were significantly decreased (*p* < 0.05). There is no significant difference between different processed products from the same origin, but the Epo levels of SHG were lower than SLG (*p* < 0.05).

### The mRNA Expression of EpoR in Hepar

**FIGURE 3:**
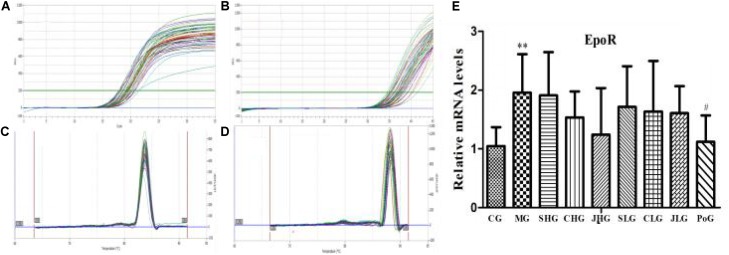
The mRNA expression of EpoR in hepar. **(A)** β-actin and **(B)** Epo are PCR amplification curve. **(C)** β-actin and **(D)** Epo are dissociation curve. **(E)** The relative mRNA levels of EpoR. CG, control group, MG, model group, SHG, Curcumae Longae Radix group, CHG, Stir-frying Curcumae Longae Radix with vinegar group, JHG, Stir-frying Curcumae Longae Radix with wine group, SLG, Curcuma phaeocaulis Radix group, CLG, Stir-frying Curcuma phaeocaulis Radix with vinegar group, JLG, Stir-frying Curcuma phaeocaulis Radix with wine group and PoG, positive group. Values were mean ± SD (*n* = 6). ^∗∗^*p* < 0.01 vs. control group; ^#^*p* < 0.05 vs. model group.

The mRNA expression of EpoR in hepar was detected by RT-PCR assay and shown in **Figure 3**. Compared with the CG, the mRNA expression of EpoR in MG was significantly increased (*p* < 0.01). After the Yujin treatment, the mRNA expression of EpoR in all groups had a downward trend, but there was no significant difference.

### The Protein Expression of EpoR in Hepar

**FIGURE 4:**
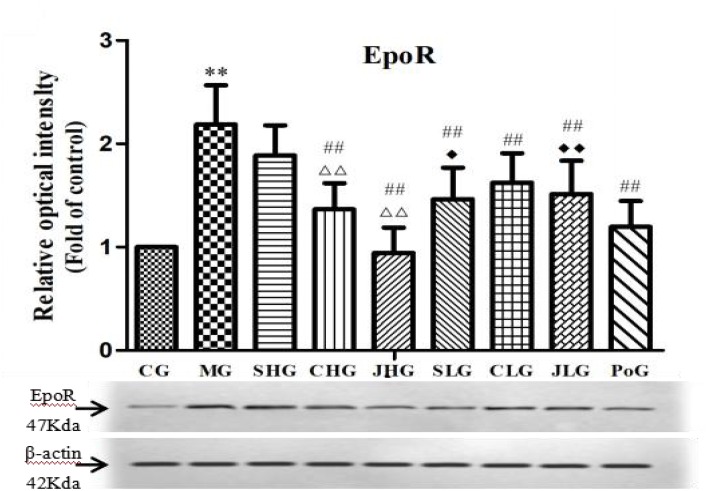
Observation for EpoR protein expression in hepar. CG, control group, MG, model group, SHG, Curcumae Longae Radix group, CHG, Stir-frying Curcumae Longae Radix with vinegar group, JHG, Stir-frying Curcumae Longae Radix with wine group, SLG, Curcuma phaeocaulis Radix group, CLG, Stir-frying Curcuma phaeocaulis Radix with vinegar group, JLG, Stir-frying Curcuma phaeocaulis Radix with wine group and PoG, positive group. Values were mean ± SD (*n* = 6). ^∗^*p* < 0.05 and ^∗∗^*p* < 0.01 vs. control group ^#^*p* < 0.05 and ^##^*p* < 0.01 vs. model group ^Δ^*p* < 0.05 and ^ΔΔ^*P* < 0.01 vs. Curcumae Longae Radix group ^
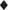
^*P* < 0.05 and ^
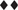
^*P* < 0.01 Curcuma phaeocaulis Radix group versus Curcumae Longae Radix group, Stir-frying Curcuma phaeocaulis Radix with vinegar group versus Stir-frying Curcumae Longae Radix with vinegar group, Stir-frying Curcuma phaeocaulis Radix with wine group versus Stir-frying Curcumae Longae Radix with wine group.

The protein expression of EpoR in hepar was detected by Western Blot assay and shown in **Figure 4**. Compared with the CG, the protein expression of EpoR in MG was significantly increased (*p* < 0.01). After the administration, all the drug groups were down regulated in different degrees, among which CHG, JHG, SLG, CLG, and JLG were highly significant difference (*p* < 0.01). There was highly significant difference among SHG, CHG, and JHG (*p* < 0.01). Meanwhile, SLG had significant difference from SHG (*p* < 0.05), and JLG had significant difference from JHG (*p* < 0.01).

### Determination of CD62p

**FIGURE 5:**
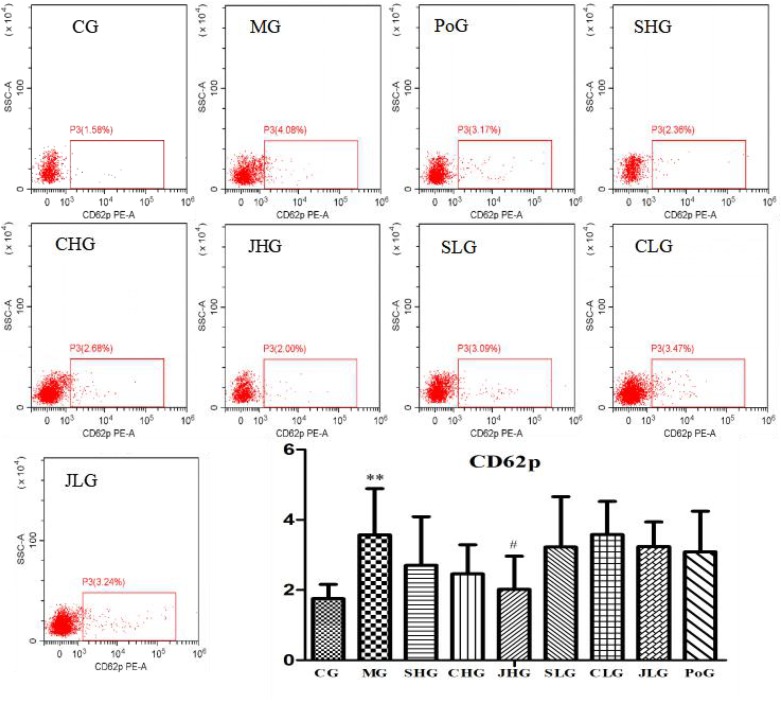
CD62p analysis using flow cytometry assay. CG, MG, PoG, SHG, CHG, JHG, SLG, CLG and JLG are images for the flow cytometry analysis of apoptosis in different groups. CG, control group, MG, model group, SHG, Curcumae Longae Radix group, CHG, Stir-frying Curcumae Longae Radix with vinegar group, JHG, Stir-frying Curcumae Longae Radix with wine group, SLG, Curcuma phaeocaulis Radix group, CLG, Stir-frying Curcuma phaeocaulis Radix with vinegar group, JLG, Stir-frying Curcuma phaeocaulis Radix with wine group and PoG, positive group. Values were mean ± SD (*n* = 6). ^∗^*p* < 0.05 and ^∗∗^*p* < 0.01 vs. control group; ^#^*p* < 0.05 vs. model group.

The differences of CD62p in the fresh liquid platelet are shown in **Figure 5**. Compared with the CG, the expression of CD62p in MG was significantly increased (*p* < 0.01). After administration, the expression of CD62p in all groups was all callback, and the best effect was JHG, with significant difference (*p* < 0.05).

## Discussion

On the basis of TCM theory, the liver stores blood and controls conveyance and dispersion. When the liver keeps the function of dispersion in order, the function of removing blood stasis is sound and the blood metabolism can be carried out smoothly. Even if there is a blood stasis factor, it is not easy to form a blood stasis syndrome. If the liver fails to control conveyance and dispersion, that means, that the function of removing blood stasis is damaged, and it is more likely to occur the blood stasis syndrom (Li, 2009). Modern medical studies have indicated that the main physiological functions of Epo are similar to the functions of liver controlling conveyance and dispersion. The Epo signaling pathway may be a suitable entry point to explore the relationship between liver storing blood and controlling conveyance and dispersion (Zhang et al., 2017). Epo, as the major regulatory factor of erythrocytogenetic (Jelkmann, 2008), can promote the proliferation of erythrocytes and prevent apoptosis of the final differentiated erythrocytes (Wang et al., 2018). Modern studies have confirmed that Epo can affect thrombosis and platelet function by improving the rate of Epo, increasing platelet count, platelet aggregation and reactivity, and enhancing the production and release of thrombopoenin (Turi et al., 1994; Bode-Boger et al., 1996; Sayinalp et al., 1998; Stohlawetz et al., 2000; Homoncik et al., 2004). This study found that the Epo level in the MG was significantly higher than that in the normal CG (*p* < 0.01), indicating that the platelet function was disorder and the blood stasis formed. This fits in with blood stasis induced by LDQS that caused by long-term anxiety, restlessness, rage, and other emotions.

Epo mainly exerts its biological function through the EpoR. In this study, RT-PCR and WB were used to analyze the expression of EpoR in rat liver tissues. It was found that EpoR protein expression and mRNA expression in liver tissues of MG increased significantly. It suggested that the Epo/EpoR pathway of model rats may be in an excited state that can lead to thrombosis by inducing platelet and activating endothelial cells. After treatment of Yujin and its processed products, the Epo level significantly decreased. But there was no significant difference between different processed products from the same origin, and the Epo level of SHG was lower than SLG. The mRNA expression and protein expression of EpoR in all groups had a downward trend. The CHG, JHG, SLG, CLG, and JLG were highly significant difference in protein expression, but there was no significant difference in mRNA expression. Meanwhile, SLG had significant difference from SHG, and JLG had highly significant difference from JHG in protein expression. It suggested that Yujin and its processed products can treatment blood stasis due to LDQS by inhibiting the Epo/EpoR pathway, reducing the proliferation of erythrocyte and platelet activation, and lowering the life span of red blood cells.

Platelet activation is involved in the occurrence and development of blood stasis syndrome (Fu, 2016). The increased expression of platelet activation molecules and the aggregation of platelets will increase the possibility of thrombosis. The body is more likely to have blood stasis syndrome (Zhu et al., 2016). CD62p is a membrane glycoprotein located in the special platelet granules and the Weibel-Palade corpuscle of the vascular endothelial cells. When the platelets are stimulated, it is rapidly expressed on the surface of the platelet membrane, which mediates the adhesion of platelets and vascular endothelium to the inflammatory cells and the adhesion of platelets, and promotes fibrin deposition and thrombosis to form (Qi and Wang, 2014). CD62p is currently one of the most specific indicators that can reflect platelet activation (Bonacina et al., 2016; Sambola et al., 2016). The results showed that, compared with CG, the expression of CD62p in the MG increased significantly, indicating that the platelet activation was enhanced, which was consistent with the blood stasis syndrome. After the administration, the expression of CD62p in each group had a callback. The effect of HSYJ was the best, which showed that the mechanism of promoting blood circulation for removing blood stasis in Yujin may lie in inhibiting the activation of platelets. The effect of HSYJ on each index was better than LSYJ, and Yujin stir-frying with wine was better than the other processed products. It is proved that the traditional theory that the effect of HSYJ is good and stir-frying with wine can strengthen the effect of promoting blood circulation for removing blood stasis.

## Conclusion

In this study, we choose the Epo/EpoR pathway and platelet activation index closely related to clinical blood stasis syndrome as the research point to discuss the mechanism and traditional processing theory of Yujin. The results indicate that the mechanism of promoting blood circulation for removing blood stasis in Yujin may be to inhibit the activation and aggregation of platelets by intervening the Epo/EpoR pathway and down regulating CD62p. Stir-frying with wine can enhance this effect. This study is important for correctly choosing the corresponding processed products in clinical of TCM and ensuring the effectiveness of clinical medication.

## Ethics Statement

This study was conducted in strict accordance with the recommendations of the Guide lines for the Care and Use of Laboratory Animals of the Ministry of Science and Technology of China. The protocol was approved by the Committee of Animal Care of Chengdu University of Traditional Chinese Medicine (Chengdu, China).

## Author Contributions

ZC overall responsibility for the manuscript. WL, LQ, HZ, YZ, and XZ performed the experiments. LH revised the manuscript and polished the language. CH involved in the design of the research.

## Conflict of Interest Statement

The authors declare that the research was conducted in the absence of any commercial or financial relationships that could be construed as a potential conflict of interest. The reviewer YG and handling Editor declared their shared affiliation.

## Abbreviations

ELISA: enzyme-linked immunosorbent assay
Epo: erythropoietin
EpoR: erythropoietin receptor
FCM: flow cytometry
HSYJ: Curcumae Longae Radix
LDQS: Qi stagnation due to Liver depression
LSYJ: Curcuma phaeocaulis Radix
RT-PCR: real-time PCR
TCM: traditional Chinese medicine
WB: western blotting
Yujin: Curcumae Radix
